# Controlling Temper

**Published:** 1878-07

**Authors:** 


### CONTROLLING TEMPER.

Fools, lunarians, the weak-minded, and the ignorant, are irascible, impatient, and of ungovernable temper: great hearts and wise, are calm, forgiving, and serene.

The most imperturbable and the ablest disputer of his age was the Scotchman, Henderson. When a glass of water was thrown in his face by the ungovernable rage into which an antagonist had allowed himself to be thrown by the anticipation of inevitable defeat, the Scotchman calmly wiped his dripping cheeks, and remarked with a smile, "That is a diversion; let us proceed with the argument."

It is said of one of the ablest men of a past century, that, having completed the manuscript of a work which he had been preparing for several years, he left his room for a few moments to find, on returning, that a favorite little dog had, in his absence, turned over the candle, and reduced his writings to ashes; on observing which, he exclaimed, "Oh! Diamond, little dost thou know the injury thou hast done ;" and immediately set about the reparation of the damages.

Philip the Second, after having sat up to a late hour in the night to complete some important state papers, waked up one of his drowsy secretaries, who was so flurried at this breach of duty, that he dashed the contents of the inkstand over the manuscript, instead of the sandbox. "It would have been better to have used the sand," was royalty's remark, on sitting down to the reproduc-of the document.

Washington, when high in command, provoked a man to knock him down. The next day he sent for the person to appear at head-quarters, and asked his pardon! for, in reviewing the incidents of the case, he found that he was himself at fault. A magnanimity only possible to a truly great mind; but it is a magnanimity, a self-control, a mastery of temper, which it is a nobility to strive for.

